# Hijacking host metabolism: PstEXLX1 suppresses *Triticum aestivum* defense responses by targeting a formate dehydrogenase

**DOI:** 10.1093/plphys/kiaf093

**Published:** 2025-03-20

**Authors:** Marcella Alves Teixeira, Mamoona Khan

**Affiliations:** Department of Plant Pathology, Washington State University, Pullman, WA 99163, USA; Department of Plant Pathology, Institute of Crop Science and Resource Conservation (INRES), University of Bonn, Bonn 53115, Germany

Caused by *Puccinia striiformis* f. sp. *tritici* (Pst), the wheat stripe rust can lead to annual losses from 4 to 5 billion USD (Figueroa et al. 2018). As an obligate biotroph, Pst relies on a large repertoire (over 2,000; Wang et al. 2023) of secreted proteins, called effectors, that may manipulate host cellular processes to promote infection and suppress immunity. However, only a few of them are functionally characterized. Effector proteins can have specific domains that determine their function and interactions with host components, such as RxLR-motif in oomycetes (Boutemy et al. 2011) and MAX-domain in fungal pathogens (de Guillen et al. 2015). Recently, effectors with expansin-like domains have gained attention for their role in modulating plant immunity, such as RsEXLX1 from *Ralstonia solanacearum* (Tancos et al. 2018) and HaEXPB2 from *Heterodera avenae* (Liu et al. 2016). Unlike plant expansins, these effectors may lack enzymatic activity but still contribute to cell wall modification and immune suppression by unknown means.

Formate dehydrogenases (FDHs) are NAD-dependent enzymes that catalyze the oxidation of formate to carbon dioxide (Hourton-Cabassa et al. 1998). They are found in various organisms, including bacteria, fungi, and plants and play a crucial role in cellular metabolism and redox balance. In plants, FDHs are primarily localized in mitochondria and are implicated in responses to abiotic stresses, particularly oxidative stress, with recent studies highlighting their involvement in plant immunity. For instance, pepper mitochondrial FDH1 has been shown to regulate cell death and defense responses against bacterial pathogens (Choi et al. 2014). In *Arabidopsis thaliana*, FDH is upregulated in response to bacterial pathogens and knockout mutants show compromised defense responses (Lee et al. 2022). Is it hypothesized that pathogens use expansin-like effectors due to its cell wall extension activity (Liu et al., 2016), to manipulate or mimic the hosts biological processes, or to modulate biological processes, such as root pathogens attachment (Tancos et al., 2018).

In this issue of *Plant Physiology*, Lu et al. (2025) identified and characterized an expansin-like effector, called PstEXLX1, by using a screen to suppress BAX (BCL2-associated X protein)—induced programmed cell death in *Nicotiana benthamiana*. The study highlights the role of PstEXLX1 in modulating plant defense responses and its impact on wheat susceptibility to stripe rust.

Lu et al. (2025) first showed that PstEXLX1 is a secreted protein and that its transcripts peak at 12 and 24 h after wheat inoculation, suggesting its relevance in early pathogenicity. The authors then used a host-induced gene silencing approach and showed that *PstEXLX1* silencing by transgenic wheat lines led to a significant reduction in Pst infection. Consistently, *PstEXLX1* overexpression in wheat resulted in increased virulence. The overexpression lines were also compromised in Pattern-Triggered Immunity responses, as evidenced by reduced chitin-induced reactive oxygen species (ROS) burst and marker gene expression.

One of the key discoveries of this study is that PstEXLX1 interacts with itself through its expansin-like domain, and this dimerization increases its protein stability. Additionally, in a yeast 2-hybrid screen, the authors identified a *Triticum aestivum* formate dehydrogenase (TaFDH1) as a potential interactor of PstEXLX1. Further, by using a series of molecular and biochemical approaches, Lu et al. (2025) confirmed that PstEXLX1 interacts and targets TaFDH1 to inhibit its activity. Consistently, the TaFDH1 overexpression lines showed increased resistance against Pst and increased defense responses as shown by enhanced chitin-induced ROS burst and defense marker gene expression. Intriguingly, they revealed a novel localization of TaFDH1 in plant cytoplasm, rather than in the mitochondria, as previously observed for other FDHs (Choi et al. 2014; Vigani et al. 2017; Lee et al. 2022; Marzorati et al. 2023; Fig. 1).

**Figure 1.**
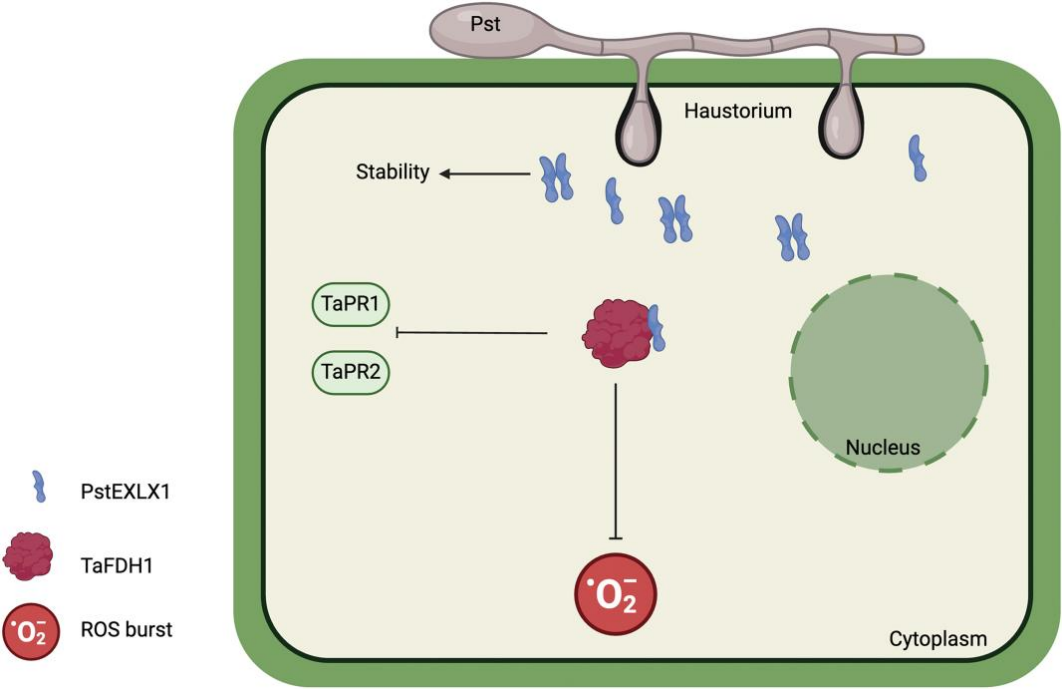
Diagram demonstrating *PstEXLX1* suppression of *Triticum aestivum* defenses. Pst secretes PstEXLX1 into plant cells, where it can self-associate to increase its stability inside plant cells. Additionally, PstEXLX1 targets TaFDH1 to inhibit its activity and disrupt defense responses, such as ROS burst and expression of defense-related genes. Both interactions take place in the cytoplasm. Figure adapted from Lu et al. (2025) and Created in BioRender. Teixeira, M. (2025) https://BioRender.com/y77u325.

To summarize, the findings by Lu et al. (2025) offer new insights into the molecular interactions between *Triticum aestivum* and Pst. The authors proposed a model in which the Pst effector PstEXLX1 enhances pathogen virulence and suppresses wheat immune responses by directly inhibiting TaFDH1 function. Although TaFDH1 is shown to be a positive regulator of *Triticum aestivum* defense, the precise mechanism remains unclear. Future studies should explore whether TaFDH1 activity contributes to other defense pathways beyond ROS production. Additionally, understanding how PstEXLX1 evolved to specifically target TaFDH1 could provide deeper insights into host-pathogen coevolution.

## Data Availability

No new data were generated or analysed in support of this research.
